# Brain Metabolic Alterations in Rats Showing Depression-Like and Obesity Phenotypes

**DOI:** 10.1007/s12640-019-00131-w

**Published:** 2019-11-28

**Authors:** Katarzyna Głombik, Jan Detka, Joanna Góralska, Anna Kurek, Bogdan Solnica, Bogusława Budziszewska

**Affiliations:** 1 Department of Experimental Neuroendocrinology, Maj Institute of Pharmacology Polish Academy of Sciences, Smętna 12, 31-343 Kraków, Poland; 2 Department of Clinical Biochemistry, Jagiellonian University Medical College, Kopernika 15A, 31-501 Kraków, Poland

**Keywords:** Depression, High-fat diet, Glucose, Oxidative phosphorylation, Mitochondria, Brain

## Abstract

Current data suggest an important role of brain metabolic disturbances in the pathogenesis of depression and obesity, diseases that frequently co-occur. Our aim was to determine whether there are changes in markers characterizing glucose metabolism in prenatal stress (PS; animal model of depression), in rats fed a high-fat diet (HFD), and especially in the model of depression and obesity co-occurrence. The changes in glucose-6-phosphate, glycogen, glucose transporters (GLUT1, GLUT4), glucagon-like peptide-1 receptor (GLP-1R), and mitochondrial complexes levels in the frontal cortex and/or hippocampus were observed. In the case of the coexistence of depression and obesity, the most important changes were (1) the decrease in the membrane form of GLUT4, which may suggest weaker insulin action in the frontal cortex, and (2) the diminished GLP-1R, which could cause neurodegenerative changes in the hippocampus. However, presented results suggested that HFD weakened the PS effect of uncoupling oxidative phosphorylation in the frontal cortex.

## Introduction

Epidemiological studies indicate a frequent comorbidity of major depression and obesity. These diseases affect an increasing number of people and have become a serious medical and social burden (Renn et al. 2011; Lopresti and Drummond 2013). People with diabetes type 1 or type 2 or obesity suffer from depression twice as often as people without this disease (de Groot et al. 2001) and, on the other hand, about doubled increased risk of obesity was found in patients with bipolar disorders than in control people (Goldstein et al. 2011). Moreover, obese people with bipolar disorder have longer and more severe depressive episodes and the therapeutic effects of some antidepressant drugs are slower and weaker (Woo et al. 2016). The reasons for this mutual relationship between obesity and depression are unknown, but following conditions are considered as possible mediators: unhealthy dietary patterns, low physical activity, sleep disorders, metabolic disorders, mitochondrial disturbances, increased inflammation, oxidative stress, neuroprogression, early-life trauma, and hypothalamus–pituitary–adrenal (HPA) axis disturbances (Lopresti and Drummond 2013). The results of many studies show that chronic stress associated with mild activation of HPA axis may be an important factor in the pathogenesis of depression and that prolonged stress combined with an increased consumption of a high-fat diet (HFD) are key factors in the development of obesity (Ayanian et al. 2009). In contrast to the stress occurring in adults, the effects of which are usually short term and disappear after the end of the stress factor action, stress in the prenatal or early postnatal period often permanently changes the HPA axis activity and increases the risk of depression, metabolic, and cardiovascular disorders (Bose et al. 2009; Murgatroyd et al. 2009; Szymańska et al. 2009; Reynolds 2010).

Accumulating evidence suggests that metabolic disturbances in brain structures involved in mood regulation may be involved in the pathogenesis of depression. In addition, obesity, often resulting from consumption of high-fat diet, causes or intensifies metabolic changes not only in the periphery but also in the brain (Hryhorczuk et al. 2013; Lopresti and Drummond 2013). Since glucose is practically the only energy source for brain cells, the disturbances in its metabolism may be key factors in the pathogenesis of both obesity and depression. Although the glucose metabolism in the brain is not yet fully understood, most of this carbohydrate in the human brain is oxidized to produce large amounts of ATP necessary to maintain proper brain cells function, including neuronal transmission. Glycolysis occurs mainly in astrocytes and lead principally to the formation of lactate. Some amount of lactate may be taken up by neurons, converted to pyruvate and then metabolized via the Krebs cycle through oxidative phosphorylation (Kasischke et al. 2004; Goyal et al. 2014). The oxidative phosphorylation process occurs mainly in neurons, cells which need the greatest amount of energy stored in ATP. In addition to glycolysis, in the brain, glucose is metabolized also in the pentose cycle, a major source of NADPH needed for reactive oxygen species removal. Brain glycogen is stored mainly in astrocytes and when the energy demand increases, it is metabolized to lactate and may be transported to neurons. However, the level of glycogen in the brain is low compared to peripheral tissues and its level usually correlates with neural activity. With regard to the regulation of glucose metabolism in the brain, particular interest is focused on the role of endocrine factors, such as glucocorticoids, insulin, and incretin hormones (Gerozissis 2008; Duarte and Moreira 2012; Ghasemi et al. 2013).

The adverse effect of glucocorticoids on peripheral metabolism, such as an increase in blood glucose level and induction of insulin resistance have been identified, but the metabolic action of this hormone in brain structures is still poorly understood. However, it is known that chronic unpredictable mild stress evokes glucose intolerance and impairs hypothalamic insulin signaling in rats (Pan et al. 2013). Long-term administration of corticosterone or removing the source of its production (adrenalectomy) changes the activity of enzymes that metabolize glucose and glycogen in certain structures of the cerebral cortex and the hippocampus (Plaschke et al. 1996; Hoyer and Lannert 2008). Our previous research also demonstrated changes in the concentration and metabolism of glucose in the frontal cortex and hippocampus of prenatally stressed rats, one of the animal models of depression (Detka et al. 2014, 2015). However, these changes, while only slight under basic conditions, were much more significant under the influence of aversive factors, such as glucose loading or acute stress, acting on these animals as adults, which is consistent with the hypothesis that stress in this period of life intensifies the effects of unfavorable factors acting later in life.

The action of brain insulin and the incretin hormone glucagon-like peptide 1 (GLP-1) on the regulation of feeding behavior and glucose homeostasis is connected and so far has mainly been studied in the hypothalamus; however, it is known that these hormones enhance neuronal survival in other brain structures, mainly the hippocampus, and improve cognitive processes (Detka et al. 2013). Therefore, as observed in both depression and obesity, disturbances in synaptic plasticity, and cognitive dysfunction may result from changes in the levels or action of both insulin and GLP-1; however, it is unclear whether this effect is due to their direct action or related to their effects on metabolic processes in these regions of the brain. Normal metabolism, whose main substrate in the brain is glucose, determines the maintenance of the membrane potential and the proper function of neurons, so metabolic changes can be crucial not only in mental illness but also in the cognitive disturbances observed in other diseases.

Because current studies indicate the occurrence of metabolic disturbances in the brain in depression, type II diabetes, and obesity, the aim of the present study was to determine whether there are changes in selected markers characterizing glucose metabolism and whether they are accompanied by disturbances in insulin, GLP-1, and their receptors levels in an animal model of depression and in rats fed with a high-fat diet. Because the high-fat diet influences the effects caused by stress in a different way, the aim of the present study was also to assess the impact of HFD on the prenatal stress effect. Additionally, since prenatal stress was used in the present study as an animal model of depression and not all animals evoked depression-like behavioral changes, (which were verified by an increase in immobility time in the Porsolt test), prenatally stressed rats were divided into prenatal stress-reactive (PS-R) and prenatal stress-nonreactive (PS-NR) groups. Despite the use of identical stressors, not all animals show depression-like behavior; such a situation has also been observed in other animal models of this disease (Kolasa et al. 2014). Selected metabolic markers were determined in the hippocampus and frontal cortex, since changes are mainly observed in those structures in depression and the adverse effects of prenatal stress occur in these structures as well.

## Materials and Methods

### Animal Treatment

#### Animals

Sprague-Dawley rats (Charles River Laboratories, Hamburg, Germany) with an initial weight of 200–250 g were maintained at a room temperature of 22 ± 2 °C on a 12-h light/dark cycle (lights on at 6:00 am) with food and water available ad libitum. All experiments were performed according to the National Institutes of Health Guide for the Care and Use of Laboratory Animals and were approved by the Local Ethics Committee in Krakow, Poland (permission no. 1144/2015).

#### Prenatal Stress Procedure

One week after arrival, vaginal smears from females were collected daily to determine the phase of the estrous cycle. During *proestrus*, female rats were placed with males for 12 h. Next, vaginal smears were tested for the presence of sperm. Pregnant females were then randomly assigned to the control and stress groups. Prenatal stress was applied as previously described (Morley-Fletcher et al. 2003; Glombik et al. 2017). Briefly, pregnant female rats were subjected to three daily sessions of immobility stress (starting at 09:00 am, 12:00 pm, and 05:00 pm). During these times, animals were placed in plastic cylinders (7/19 cm) and exposed to a bright light (2 × 150 W bulbs) for 45 min. The stress sessions were performed from day 14 of pregnancy until delivery. Control pregnant females were left undisturbed in their home cages. Twenty-one days after birth, male offspring from litters containing 10–14 pups with a comparable number of males and females were separated for further experiments (1–2 rats from the same litter were used in the study). The rats were housed in groups of five per cage under standard conditions until 3 months of age.

#### Forced Swim Test in Rats

The 3-month-old animals were individually subjected to two trials in which they were forced to swim in a cylinder filled with water (23 °C) up to a height of 35 cm. The first trial (pretest) lasted 15 min, and the second trial lasted 5 min, during which the total duration of immobility was measured. Then, the prenatally stressed rats were divided into groups: prenatal stress-responsive and prenatal stress-nonresponsive groups.

- Prenatal stress reactive (PS-R): rats in which the total immobility time was significantly higher than the immobility time of the control group. In these animals, the immobility time was by > 1 SD higher than the mean of the control rats.
- Prenatal stress nonreactive(PS-NR): rats in which no significant differences in immobility time were present compared to the immobility time of the control group.

Forced swim test was repeated after 16 weeks of diet.

#### Diet

Rats from the prenatally stressed groups and the control group were transferred in groups of four animals to one cage and then divided into two dietary groups: (i) a group fed with standard laboratory chow (STD) containing 13.6% kcal from fat (61.5% kcal from carbohydrates; 24.9% kcal from protein) and (ii) a group fed with a high-fat diet (HFD) containing 45% kcal from unsaturated fat (lard) (35% kcal from carbohydrates; 20% kcal from protein). Both chows were purchased in the laboratory chow producer “Wytwórnia Pasz Morawski” Kcynia, Poland. The animals were fed a standard or high-fat diet for 16 weeks, and during this time, they were weighed every 7 days.

**Fig. 1:**
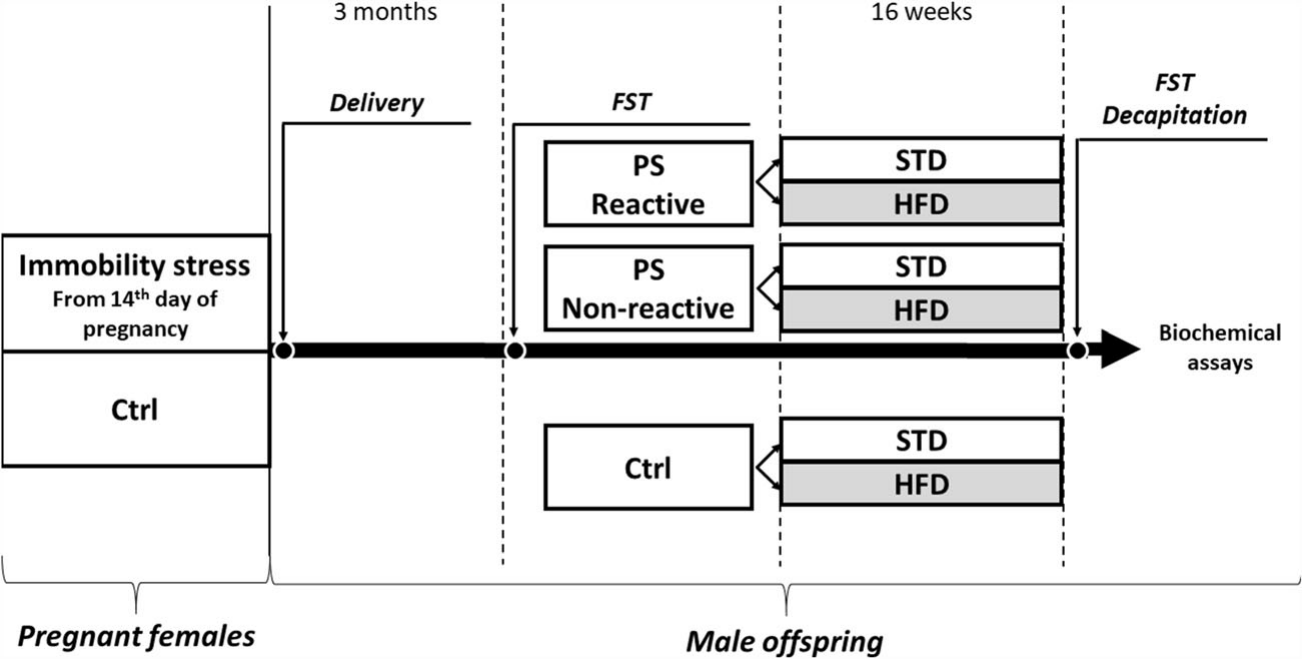
The timeline showing experimental design. Control (Ctrl) and prenatally stressed (PS) male rats were subjected to forced swim test (FST) at 3 months of age. After FST, PS rats were divided into prenatal stress-reactive (PS-R) and prenatal stress-nonreactive (PS-NR) on the basis of total immobility time in the test. Subsequently, the animals from both stressed groups and control rats consumed standard (STD) or high-fat diet (HFD) for 16 weeks. After this time period, FST was conducted once again, animals were sacrificed, and brain structures were isolated for biochemical assays

The timeline showing the experimental design is illustrated in Fig. 1.

### Biochemical Analysis

Two independent animal experiments were performed. All biochemical experiments were carried out with single repetition of each individual sample, exactly the same conditions for every sample, regardless of the type of animal treatment.

#### Tissue Collection

The animals were sacrificed under nonstressful conditions by rapid decapitation (rats were not fasted before sacrifice). The brains were rapidly removed, and the hippocampus and frontal cortex were dissected on ice-cold glass plates. Tissues were frozen on dry ice and stored at − 80 °C till the biochemical assays.

**Scheme 1:**
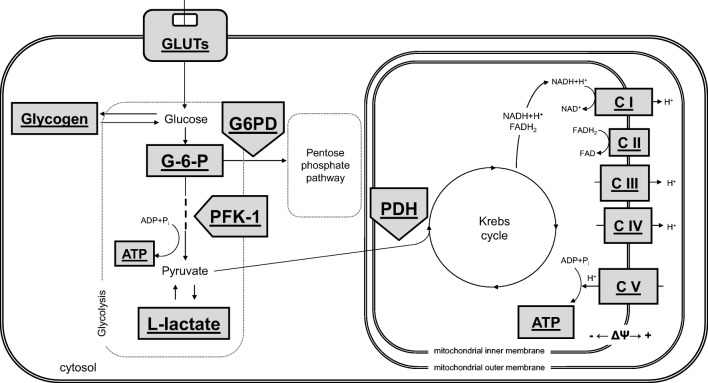
The simplified diagram of cellular glucose metabolism, illustrating key metabolites and enzymes of glycolysis, pentose phosphate pathway, Krebs cycle, and oxidative phosphorylation, which were measured in the experiment

Key metabolites and enzymes of glycolysis, pentose phosphate pathway, Krebs cycle, and oxidative phosphorylation determined in present study are shown on Scheme 1.

#### Glycogen Assay

Glycogen concentration in the frontal cortex and hippocampus was measured with an enzymatic method using a fluorimetric assay kit (#K646-100; BioVision, USA). Samples were homogenized in 5 volumes of redistilled water, boiled for 5 min at 96 °C to inactivate the enzymes and centrifuged at 20,000×*g* for 20 min. Forty microliters of each supernatant was transferred to a 96-well plate along with blanks and standards (0.2, 0.4, 0.6, 0.8, and 1.0 μg per well). In the selected assay, glycogen is hydrolyzed to glucose (by glucoamylase), which is subsequently oxidized and reacts with an OxiRed probe to produce a fluorophore. Fluorescence intensity was measured at excitation and emission wavelengths of 535 and 590 nm, respectively, with a fluorometer (Tecan Infinite 200 Pro, Switzerland). The concentration of glycogen was calculated by subtracting the background fluorescence (amount of glucose in unhydrolyzed samples) from the fluorescence intensity of the samples after hydrolysis. Glycogen levels were then calculated from the standard curve and displayed as μg/mg of protein.

#### Glucose-6-Phosphate Assay

The levels of glucose-6-phosphate (G-6-P) in the examined brain structures were determined with enzymatic methods using colorimetric assay kits (#K657-100, BioVision, USA). Brain tissues were homogenized in PBS, centrifuged at 20,000×*g* for 20 min at 4 °C, and deproteinized using a perchloric acid/KOH protocol (#K808-200; BioVision, USA). Fifty-microliter aliquots of samples were transferred to 96-well plates, mixed with 50 μl of Reaction Mix and incubated at room temperature for 30 min. The absorbance was measured at *λ* = 450 nm (Tecan Infinite 200 Pro spectrophotometer, Switzerland). The concentration of G-6-P was calculated from the standard curve and displayed as nmol/mg of protein.

#### Determination of L-Lactate

To measure the concentration of L-lactate in selected brain structures, tissues were homogenized in PBS, centrifuged (20,000×*g*; 20 min.; 4 °C) and deproteinized using a perchloric acid/KOH protocol (#K808-200; BioVision, USA). The level of lactate in the samples was measured with a colorimetric assay kit (#K607-100, BioVision, USA). Seven-microliter aliquots of the samples were transferred to a 96-well plate and mixed with 50 μl of Reaction Mix. After a 30-min incubation, the absorbance was measured at *λ* = 570 nm. The concentration of lactate in each sample was calculated from the standard curve and finally displayed as nmol/mg of protein.

#### Enzyme-Linked Immunosorbent Assay (ELISA)

The concentrations of phosphofructokinase, glucose transporter (GLUT1), GLP-1, GLP-1 receptor (GLP-1R), GLP-2 receptor (GLP-2R), insulin, and active (phosphorylated at tyrosine 1162/1163) and total insulin receptor (phospho-IR and IR) in selected brain structures were determined by using an ELISA method with commercially available assay kits (GLUT1: SEB185Ra, USCN Life Science Inc.; Phosphofructokinase: SED406Ra, USCN Life Science Inc.; GLP-1: EGLP-35K, Merck Millipore; GLP-1R: MBS2031967, MyBioSource; GLP-2R: MBS 9321753, MyBioSource; insulin: RI-13K, Merck Millipore; phospho-IR: 17-484, Merck Millipore; IR: 17-483, Merck Millipore p-IRS: 17-459 Merck Millipore). Each individual sample was transferred to a precoated 96-well ELISA plate along with the appropriate standards, blanks, and positive controls. The concentrations of selected markers were calculated from the standard curve and subsequently divided by the protein content in a given sample.

#### Determination of Pyruvate Dehydrogenase Activity

Pyruvate dehydrogenase activity was measured with a colorimetric assay kit (#K679-100, BioVision, USA). Brain structures were homogenized in 4 volumes of assay buffer, centrifuged at 10,000×*g* for 5 min at 4 °C. Ten microliters of each supernatant was transferred in to a 96-well assay plate along with the appropriate standards and subsequently mixed with 50 μl of Reaction Mix. The plate was then placed in a spectrophotometer (Tecan Infinite M200 Pro, Switzerland). The absorbance was measured at 37 ° C every 10 min for a total time of 1 h, *λ* = 450 nm. Activity of pyruvate dehydrogenase was then calculated and finally displayed as nmol/min/mg of protein.

#### Determination of Glucose-6-Phosphate Dehydrogenase Activity

The activity of glucose-6-phosphate dehydrogenase in the frontal cortex and hippocampus was measured using a colorimetric assay kit (MAK015, Sigma-Aldrich, USA). Brain tissues were homogenized in four volumes of PBS and centrifuged at 15,000×*g* (10 min, 4 °C). Forty-microliter aliquots of the supernatants were transferred to a 96-well plate and mixed with 50 μl of Master Reaction Mix. The absorbance was measured two times at a wavelength of *λ* = 450 nm: first, immediately after substrate addition and the second time, after 20 min in order to determine reaction kinetics. The activity of the enzyme was calculated from the standard curve and finally displayed as mU/mg of protein.

#### Isolation of Mitochondria-Enriched Membrane and Cytosolic Fractions

To determine the activity and the amount of selected mitochondrial enzymes, as well as measure the translocation of GLUT4 to the cell membrane, mitochondria-enriched membrane fraction was isolated from the frontal cortex and hippocampus according to the procedure described by Wernicke et al. (2010). Briefly, brain tissues, kept on ice, were homogenized in a motor-driven Teflon-glass homogenizer in four volumes of homogenization buffer containing 5 mol/l HEPES/NaOH, pH 7.4, 320 mmol/l sucrose, and 1 mmol/ l Na^+^/EDTA with the addition of 0.5% protease inhibitor cocktail (Sigma-Aldrich, USA). After centrifugation at 1300×*g* (4 min, 4 °C), supernatants were collected. Additionally, to increase the yield, the pellet was washed twice with homogenization buffer and centrifuged at 1500×*g* (4 min, 4 °C). To collect the mitochondria, supernatants were combined and centrifuged at 17,000×*g* for 12 min at 4 °C. The obtained pellets containing mitochondria and supernatants (containing the cytosolic fraction) were rapidly frozen and stored at − 80 °C.

#### Determination of GLUT4 Concentration in Membrane and Cytosolic Fractions

Level of GLUT4 in the frontal cortex and hippocampus was measured by using the ELISA method using a specific assay kit (E2023r, Wuhan EIAab® Science Co., Ltd, China). The membrane fraction was initially suspended in PBS, sonicated in order to release membrane-bound proteins, and centrifuged at 12,000×*g* for 12 min at 4 °C. After centrifugation, supernatants were transferred to a precoated 96-well plate along with blanks and standards. The cytosolic fraction was directly aliquoted into an ELISA plate. The final concentrations of GLUT4 in both cellular fractions were displayed as ng/mg of protein.

#### Immunoblotting To Detect Expression of Phospho-Akt/Akt (in Tissue Homogenates) and Respiratory Chain Complexes (CI-CV), Neuronal Uncoupling Protein 4 (UCP4) (in Mitochondria-Enriched Membrane Fraction)

The brain structures were lysed in 2% SDS (BioShop, Canada), while the isolated mitochondria-enriched membrane fraction was lysed in PBS (containing 1% Triton X-100, 0.1% SDS, phosphate, and protease inhibitors (Thermo Fisher Scientific, USA)). Then, samples were shaken for 10 min and centrifuged for 10 min, at 12,000×*g*. Samples containing equal amounts of total protein were mixed with the gel loading buffer (Bio-Rad, Hercules, USA) in a 4:1 ratio (v/v) and incubated at 37 °C for 5 min (mitochondria-enriched fraction samples) or at 95 °C for 8 min (homogenate samples). Next, proteins were separated by SDS-PAGE (4–20% gel, Bio-Rad, USA) under constant voltage (150 V). After electrophoresis, proteins were transferred to PVDF membranes. The blots were blocked in 5% nonfat dried milk in PBS with 0.05% Tween 20 (Sigma-Aldrich, USA) or BSA for 1 h at room temperature (RT) and incubated overnight at 4 °C with phospho-Akt (Ser473) (1:2000, #4060, Cell Signaling, USA), OXPHOS Rodent WB Antibody Cocktail at a 6.0-μg/ml working concentration (ab110413, Abcam, UK) or UCP4 Polyclonal Antibody (1.0 μg/ml, PA5-69265, Thermo Fisher Scientific, USA) that had been diluted in a SignalBoost Immunoreaction Enhancer Kit (Merck Millipore, USA). The day after, membranes were rinsed 3 × 10 min in PBST (PBS with 0.05% Tween 20) and incubated at RT with horse anti-mouse/goat anti-rabbit IgG HRP peroxidase-conjugated secondary antibody (PI-2000, PI-1000; respectively, Vector Laboratories, UK) for 1 h. After washing for 4 × 10 min in TBST (Tris-buffered saline with 0.05% Tween 20), the bands were developed using BM Chemiluminescence Western Blotting Substrate (POD) (Roche, Germany). The protein pattern images were obtained using a Fujifilm LAS-1000 System (Fuji Film, Japan). The relative levels of immunoreactivity were densitometrically quantified using the Fujifilm Multi Gauge software (Fuji Film, Japan). The blots were stripped using stripping buffer containing 100 ml of Tris-HCl (pH = 6.7), 2% SDS, and 700 μl of 2-mercaptoethanol (all from Sigma-Aldrich, USA); washed three times for 10 min each in TBST; blocked; and reprobed with an antibody against β-actin (A5441, Sigma-Aldrich, USA) as an internal loading control at a dilution of 1:15,000 in a SignalBoost Immunoreaction Enhancer Kit (Merck Millipore, USA). Concerning the Akt kinase, the stripping procedure was used to detect the total form of Akt (1:3000, #9272, Cell Signaling, USA). β-Actin was determined at the same time as the phosphorylated form of Akt (after transfer, the membranes were cut to allow simultaneous incubation with two antibodies: phospho-Akt and β-actin).

#### ATP Assay

The intracellular ATP content was measured using an ATPlite™ Luminescence ATP Detection Assay System (Perkin Elmer, USA). The results were calculated as nmol ATP, adjusted for protein content, and reported as nmol ATP/g of protein (Sliwa et al. 2012).

#### Mitochondrial Respiratory Function

The respiration of brain mitochondria was measured at 37 °C by high-resolution respirometry with an Oxygraph-2k (Oroboros Instruments, Austria). Rats were sacrificed, and the frontal cortex was removed and briefly placed in tubes containing ice-cold homogenization buffer (0.25 M sucrose, 50 mM KCl, 5 mM EDTA, 1 mM sodium pyrophosphate, 5 mM MgCl_2_ (pH 7.4), Sigma-Aldrich, USA) with freshly added protease inhibitor cocktail (Thermo Fisher Scientific, USA). Tissue was homogenized 10 times with a Teflon-glass homogenizer on ice, and the homogenates were centrifuged at 1300 rpm for 10 min (4 °C). The supernatants were collected (SN1), and the pellet were resuspended in 4 volumes of homogenization buffer. Samples were centrifuged as described previously, and supernatant was collected (SN2). SN1 and SN2 were mixed and centrifuged at 9000×*g* for 15 min. Then, the pellet was resuspended carefully in 5 volumes of homogenization buffer. The protein level was measured using the BCA method (Smith et al. 1985). We used 300 μg of isolated mitochondria suspended in 2 ml of MiR05 respiration buffer (in mmol/l, EGTA 0.5, MgCl_2_·6H2O 3, K-lactobionate 60, taurine 20, KH_2_PO_4_ 10, HEPES 20, sucrose 110, fatty acid-free BSA 1 g/L, pH 7.0 with KOH, Sigma-Aldrich, USA) per Oxygraph-2k chamber for each experiment (based on preliminary experiments). Mitochondrial respiration was measured in the presence of substrates (glutamate and malate). All respiration measurements were made following this protocol: glutamate (10 mM) and malate (2 mM) without ADP (leak state); respiration assessed by the addition of 2.5 mM ADP (ADP-stimulated state, control of coupled respiration by complex I) and, next, the addition of 10 mM succinate (CI + CII OXPHOS state). Then, uncoupling control through the titration of the protonophore carbonylcyanide-4-(trifluoromethoxy)-phenylhydrazone (FCCP)-uncoupled state, optimal for 0.25 μM) was measured (Pesta and Gnaiger 2012; Sebastian et al. 2012). Finally, rotenone (1 μM) and antimycin A (2.5 μM) were added to inhibit complex I and complex II, respectively. Inhibition of respiration of uncoupled mitochondria leads to evaluation of oxygen flux due to oxidative side reactions (residual oxygen consumption, ROX). Data from high-resolution respirometry were analyzed using the DatLab 4 software (Oroboros, Austria) and are presented as O_2_ flux per mass, ROX-corrected.

Mitochondrial respiratory function was measured only in the frontal cortex in four groups: control animals fed with standard and high-fat diet and animals responding to prenatal stress (increased immobility time in the Porsolt test) receiving standard and high-fat diet.

#### Measurement of Protein Concentration

The protein concentration in tissue homogenates was measured with the bicinchoninic acid method (Smith et al. 1985) using a Pierce™ BCA Protein Assay Kit (Thermo Fisher Scientific, USA).

### Statistical Analysis

All data are presented as the mean ± SEM. The results were analyzed using the STATISTICA software. The subsequent statistical analyses used factorial ANOVA of the PS/diet to determine the effects of the examined factors. Next, Duncan’s post hoc test was applied. A *p* value < 0.05 was considered to indicate significance.

## Results

### Prenatal Stress Affected the Behavioral Parameters Measured in the Forced Swim Test

**Fig. 2:**
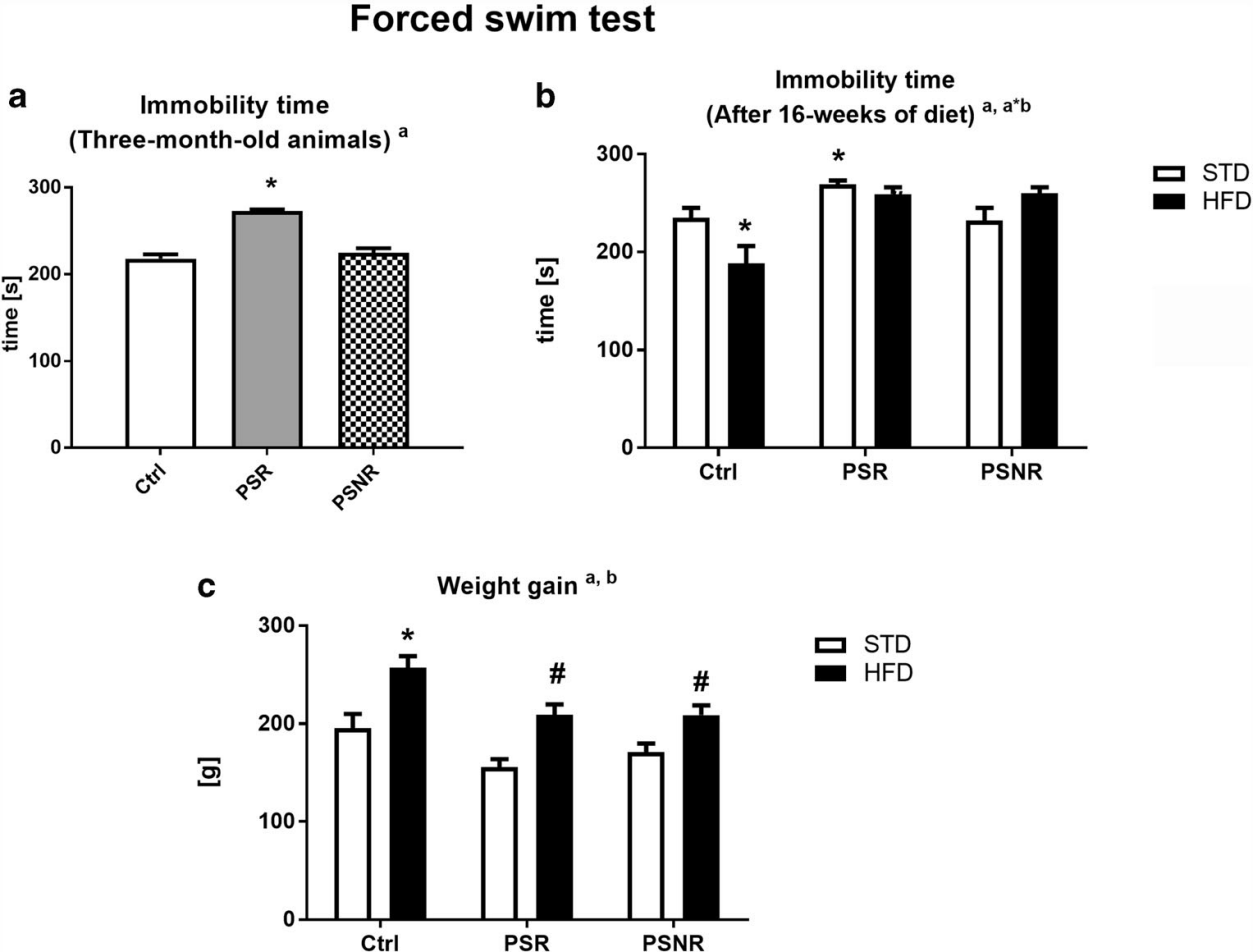
The effect of prenatal stress on immobility time (**A**) in forced swim test. *n* = 32. The effect of prenatal stress and 16-week consumption of high-fat diet on immobility time (**B**) in forced swim test. *n* = 8. The effect of 16-week consumption of high-fat diet on body weight gain of animals from control and prenatal stress-reactive (PS-R) and prenatal stress nonreactive (PS-NR) group (**C**). *n* = 14-16. The results are expressed as the means ± SEM. (a) Statistically significant effect of prenatal stress; (b) statistically significant effect of HFD; *p < 0.05 vs. control group (without prenatal stress, fed with STD); ^#^*p* < 0.05 vs. appropriate STD group

ANOVA revealed a significant effect of PS on total immobility time (*F*_2,93_ = 53.25, *p* < 0.0001) measured in the forced swim test (Fig. 2A). Furthermore, post hoc analysis showed that prenatally stress-reactive (PS-R) rats were characterized by extended total immobility time (*p* = 0.0001) in comparison to control animals, whereas prenatal stress nonreactive rats (PS-NR) displayed no significant differences in this parameter when compared to control. After the 16-week consumption of a high-fat diet, an effect of PS (*F*_2,42_ = 12.71, *p* < 0.0001) and PS/HFD interaction (*F*_2,42_ = 6.13, *p* = 0.0046) on the immobility time was showed. The post hoc test revealed that PS-R rats fed the standard diet exhibited significantly higher levels of immobility behavior (*p* = 0.0040) than Ctrl/STD animals (Fig. 2B). Additionally, Ctrl/HFD rats displayed lower levels of immobility behavior (*p* = 0.0048) in the FST than Ctrl/STD group.

### Sixteen Weeks of High-Fat Diet Increased Weight Gain

Factorial ANOVA showed the effects of PS (*F*_2,83_ = 9.28, *p* = 0.0002) and HFD (*F*_1,83_ = 32.09, *p* < 0.0001) on weight gain after 16 weeks of feeding. Groups of animals fed with HFD shown marked increase in body weight gain, when compared to appropriate control groups, which consumed standard laboratory chow (control/HFD vs. control/STD: *p* = 0.0004; PS-R/HFD vs. PS-R/STD: *p* = 0.0020; PS-NR/HFD vs. PS-NR/STD: *p* = 0.0226) (Fig. 2C).

### PS Increased Glucose-6-Phosphate and Glycogen Level in the Frontal Cortex and Hippocampus, Whereas HFD Increased Glucose-6-Phosphate in the Hippocampus

To assess metabolic changes in the examined models of depression and obesity, first, a concentration of glucose-6-phosphate, the main intracellular substrate of carbohydrate metabolism, and the content of glycogen, a compound that is an intracellular energy store, were determined.

**Fig. 3:**
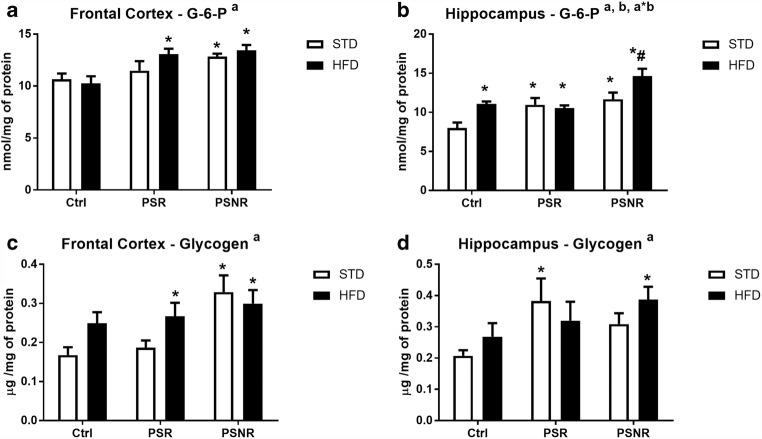
The effect of prenatal stress and high-fat diet on glucose-6-phosphate (G-6-P) (**A**, **B**) and glycogen (**C**, **D**) contents in the frontal cortex (**A**, **C**) and hippocampus (**B**, **D**). The results are expressed as the means ± SEM. (a) Statistically significant effect of prenatal stress; (b) statistically significant effect of HFD; (a*b) statistically significant effect of prenatal stress/HFD interaction. *p < 0.05 vs. control group (without prenatal stress, fed with STD); ^#^*p* < 0.05 vs. appropriate STD group. *n* = 6–8

We observed that PS (*F*_2,41_ = 13.95, *p* < 0.0001), HFD (*F*_1,41_ = 10.43, *p* = 0.0024), and PS/HFD interaction (*F*_2,41_ = 3.79, *p* = 0.0308) affected the concentration of glucose-6-phosphate in the hippocampus (Fig. 3B), whereas in the frontal cortex, only PS (*F*_2,42_ = 9.10, *p* = 0.0006) showed such an action (Fig. 3A). In the hippocampus, glucose-6-phosphate concentration was increased in all experimental groups in comparison to the control rats (control/HFD: *p* = 0.0069; PS-R/STD: *p* = 0.0073; PS-R/HFD: *p* = 0.0154; PS-NR/STD: *p* = 0.0152; PS-NR/HFD: *p* < 0.0001) and in PS-NR/HFD vs. PS-NR/STD: *p* = 0.0052, while in the frontal cortex, its level was enhanced in the PS-R animals fed with a HFD (*p* = 0.0171) and in PS-NR rats fed with STD (*p* = 0.0279), HFD (*p* = 0.0077) diet.

Factorial ANOVA showed that glycogen levels in both brain structures were changed by prenatal stress (*F*_2,41_ = 3.56, *p* = 0.0375 Hp and *F*_2,42_ = 6.63, p = 0.0032 FCx), but no effect of a HFD or the PS/HFD interaction was observed (Fig. 3C, D). We showed an elevated glycogen level in the hippocampus in PS-R rats fed with a STD (*p* = 0.0256) and in PS-NR animals receiving a HFD (*p* = 0.0245) and in the frontal cortex in PS-R rats fed a HFD (*p* = 0.0423) and in PS-NR animals fed both a STD (*p* = 0.0017) and HFD (*p* = 0.0087).

### Upregulation of GLUT1 and Downregulation of the Active Form of GLUT4 in the Frontal Cortex

Since an increase in glucose-6-phosphate and glycogen levels may be due to augmentation of glucose transport into the cell, therefore, next, the protein expression of glucose transporters GLUT1 and GLUT4 was assayed.

**Fig. 4:**
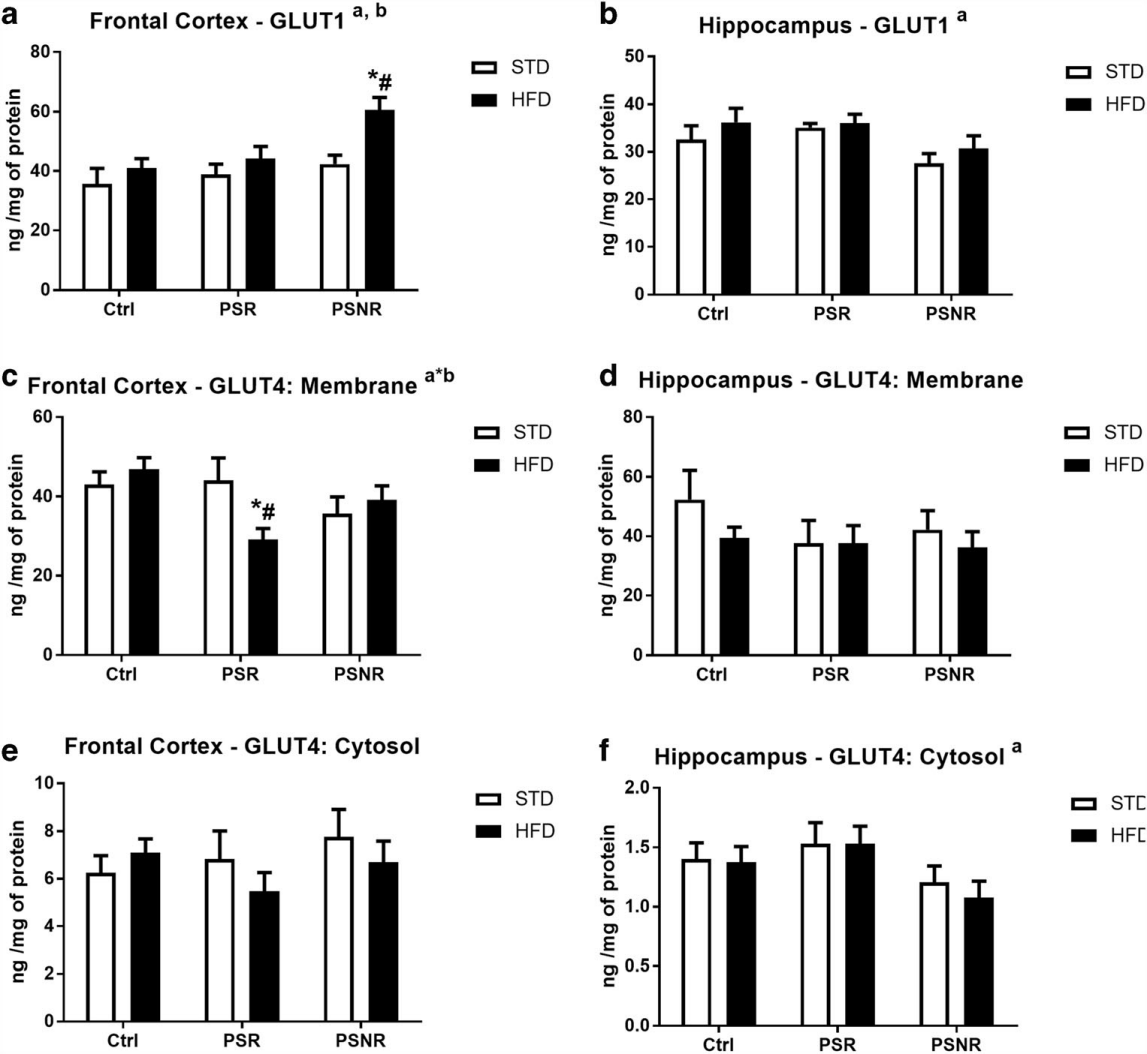
The effect of prenatal stress and high-fat diet on GLUT1 concentrations in the frontal cortex (**A**) and hippocampus (**B**), GLUT4 levels in the membrane (**C**) and cytosol (**E**) fractions of the frontal cortex, and GLUT4 levels in the membrane (**D**) and cytosol (**F**) fractions of the hippocampus. The results are expressed as the means ± SEM. (a) Statistically significant effect of prenatal stress; (b) statistically significant effect of HFD; (a*b) statistically significant effect of prenatal stress/HFD interaction. *p < 0.05 vs. control group (without prenatal stress, fed with STD); ^#^*p* < 0.05 vs. appropriate STD group. *n* = 6–8

ANOVA revealed a significant effect of PS (*F*_2,40_ = 4.32, *p* = 0.0199) on GLUT1 expression in the hippocampus and an effect of PS (*F*_2,42_ = 3.44, *p* = 0.0415) and HFD (*F*_1,42_ = 5.18, *p* = 0.0280) on the level of this transporter in the frontal cortex (Fig. 4A, B). An increase in GLUT1 in the PS-NR/HFD group vs. control/STD (*p* = 0.0037) and PS-NR/STD (*p* = 0.0233) rats in the frontal cortex was shown by the post hoc test. Despite the main effect of PS showed by analysis of variance, post hoc test showed that in the hippocampus, there was no change in the level of this transporter in any of the studied groups.

The GLUT4 transporter was measured in the membrane (active form) and cytosol fraction. In the frontal cortex ANOVA displayed a significant effect of the PS/HFD interaction (*F*_2,39_ = 3.78, *p* = 0.0316) on membrane GLUT4. Post hoc test showed a decreased level of this transporter in PS-R/HFD rats in comparison to that in control/STD (*p* = 0.0253) and PS-R/STD rats (*p* = 0.0182) (Fig. 4C). In the hippocampus, no effect of prenatal stress, HFD, or the PS/HFD interaction on GLUT4 in the membrane fraction was observed (Fig. 4D). Despite the main effect of PS (*F*_2,40_ = 3.69, *p* = 0.034) demonstrated by the analysis of variance in the hippocampus, there were no differences in the content of GLUT4 in the cytosol fraction in this structure (Fig. 4F). Moreover, it was found that none of the factors had an effect on cytosolic GLUT4 in the frontal cortex (Fig. 4E).

### PS and HFD Affected Lactate, but Not Phosphofructokinase Concentrations

The indicated increased level of glucose-6-phosphate may result from attenuation of glycolysis process, and therefore, expression of the key enzyme of this process and the main glycolysis product in the brain were determined.

**Fig. 5:**
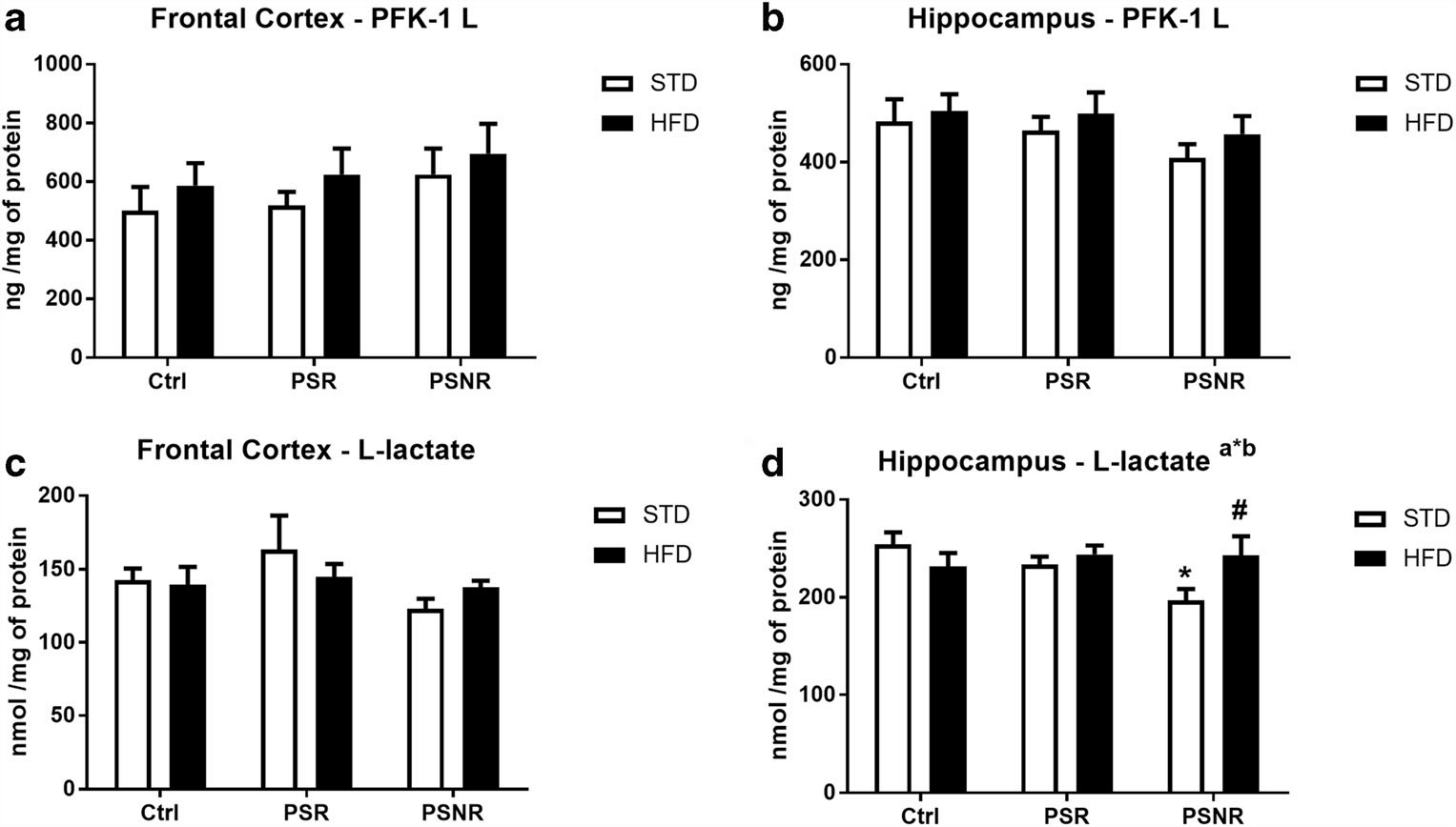
The effect of prenatal stress and high-fat diet on the expression of phosphofructokinase (PFK-1 L) in the frontal cortex (**A**) and hippocampus (**B**) and L-lactate concentrations in the frontal cortex (**C**) and hippocampus (**D**). The results are expressed as the means ± SEM. (a*b) Statistically significant effect of prenatal stress/HFD interaction. *p < 0.05 vs. control group (without prenatal stress, fed with STD); ^#^*p* < 0.05 vs. appropriate STD group. *n* = 8

It was found that none of the factors (PS, HFD, or PS/HFD interaction) had an effect on phosphofructokinase expression in the frontal cortex and hippocampus (Fig. 5A, B). Also none of the factors (PS, HFD, or PS/HFD interaction) affected the level of lactate, the main glycolytic product in the brain in the frontal cortex (Fig. 5C).

In the hippocampus, we observed an effect of the PS/HFD interaction (*F*_2,42_ = 3.48, *p* = 0.0401), but not PS and HFD, on lactate concentration. The post hoc test showed a decrease in this glycolytic marker in PS-NR/STD rats compared with control/STD rats (*p* = 0.0076) and increase in PS-NR/HFD rats vs. PS-NR/STD (*p* = 0.0259) (Fig. 5D).

### Increased Glucose-6-Phosphate Dehydrogenase Activity in the Frontal Cortex of PS-R Rats

In addition to the glycolysis process, glucose can be metabolized also in the pentose cycle that is a major source of NADPH used for reactive oxygen species removal. Weakened activity of the pentose cycle enzymes could be the cause of both the nerve cell damage by ROS and increased level of glucose-6-phosphate; therefore, the activity of the key enzyme of this process was determined.

**Fig. 6:**
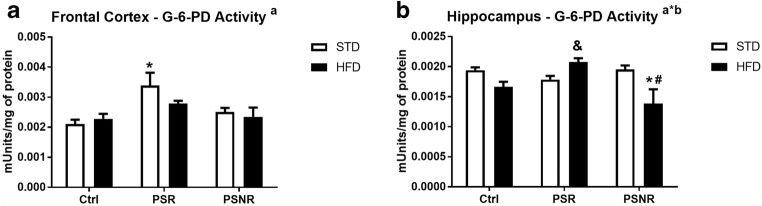
The effect of prenatal stress and high-fat diet on the glucose-6-phosphate dehydrogenase (G-6-PD) activity in the frontal cortex (**A**) and hippocampus (**B**). The results are expressed as the means ± SEM. (a) Statistically significant effect of prenatal stress; (a*b) statistically significant effect of prenatal stress/HFD interaction. *p < 0.05 vs. control group (without prenatal stress, fed with STD); ^#^*p* < 0.05 vs. appropriate STD group; ^&^*p* < 0.05 vs. control group (without prenatal stress, fed with HFD). *n* = 8

PS (*F*_2,42_ = 7.38, *p* = 0.0018) but not the HFD or PS/HFD interaction significantly affected the activity of glucose-6-phosphate dehydrogenase in the frontal cortex (Fig. 6A). Increased activity of this enzyme in the frontal cortex in prenatal stress-responsive rats fed with the STD in relation to the control/STD (*p* = 0.0013) group was shown by further analysis. In the hippocampus, PS/HFD interaction (*F*_2,42_ = 7.42, *p* = 0.0017) was indicated and the activity of this enzyme was lower in the PS-NR rats fed with the HFD compared to the control/STD (*p* = 0.0024) and PS-NR/STD groups (*p* = 0.0022). The activity of glucose-6-phosphate dehydrogenase in PS-R/HFD was higher in comparison to control /HFD group (*p* = 0.0253).

### HFD Decreased Activity of Pyruvate Dehydrogenase in the Hippocampus

To check the possibility that increased level of glucose-6-phosphate resulted from Krebs cycle attenuation, activity of pyruvate dehydrogenase, the key enzyme responsible for placing the products of glycolysis to the Krebs cycle, was determined.

**Fig. 7:**
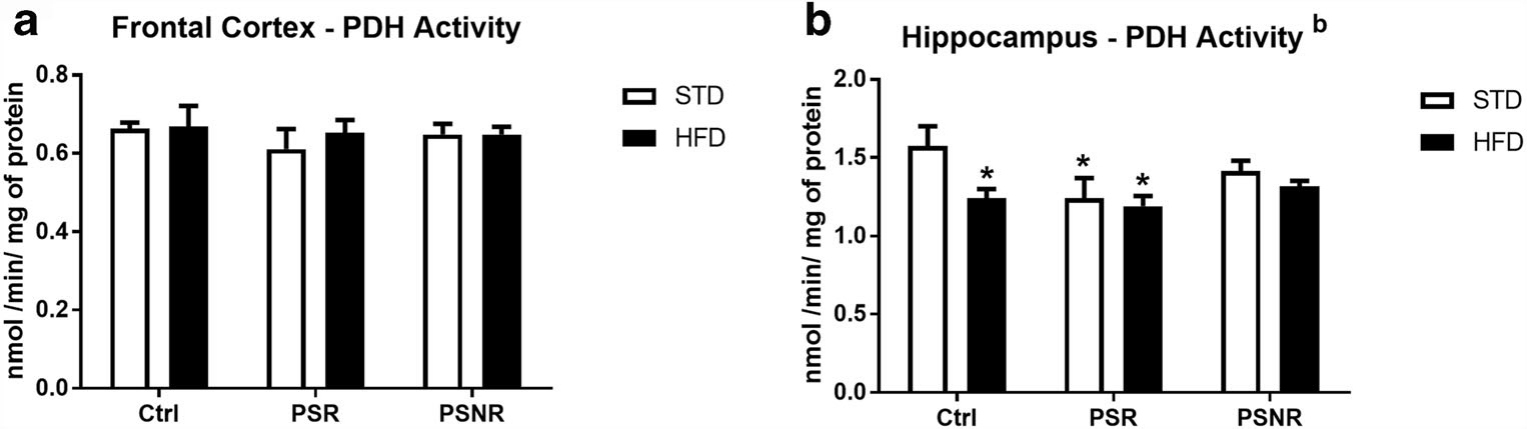
The effect of prenatal stress and high-fat diet on the activity of pyruvate dehydrogenase (PDH) in the frontal cortex (**A**) and hippocampus (**B**). The results are expressed as the means ± SEM. (b) Statistically significant effect of HFD. *p < 0.05 vs. control group (without prenatal stress, fed with STD). *n* = 8

ANOVA showed no effect of PS, HFD, and their interaction on the activity of pyruvate dehydrogenase, one of the first important enzymes in the Krebs cycle, in the frontal cortex (Fig. 7A). In the hippocampus, HFD (*F*_1,42_ = 5.08, *p* = 0.0295) affected the activity of this enzyme (Fig. 7B). Pyruvate dehydrogenase activity was decreased in the control/HFD group (*p* = 0.0184) and in prenatal stress-responsive animals fed with STD (*p* = 0.0169 ) or HFD (*p* = 0.0078).

### Upregulation of Respiratory Chain Complexes Occurred Mainly in the Frontal Cortex

After the estimation of the glycolysis process and the Krebs cycle, the next stage of metabolism, i.e., oxidative phosphorylation was assessed by determining the expression of respiratory chain complexes.

**Fig. 8:**
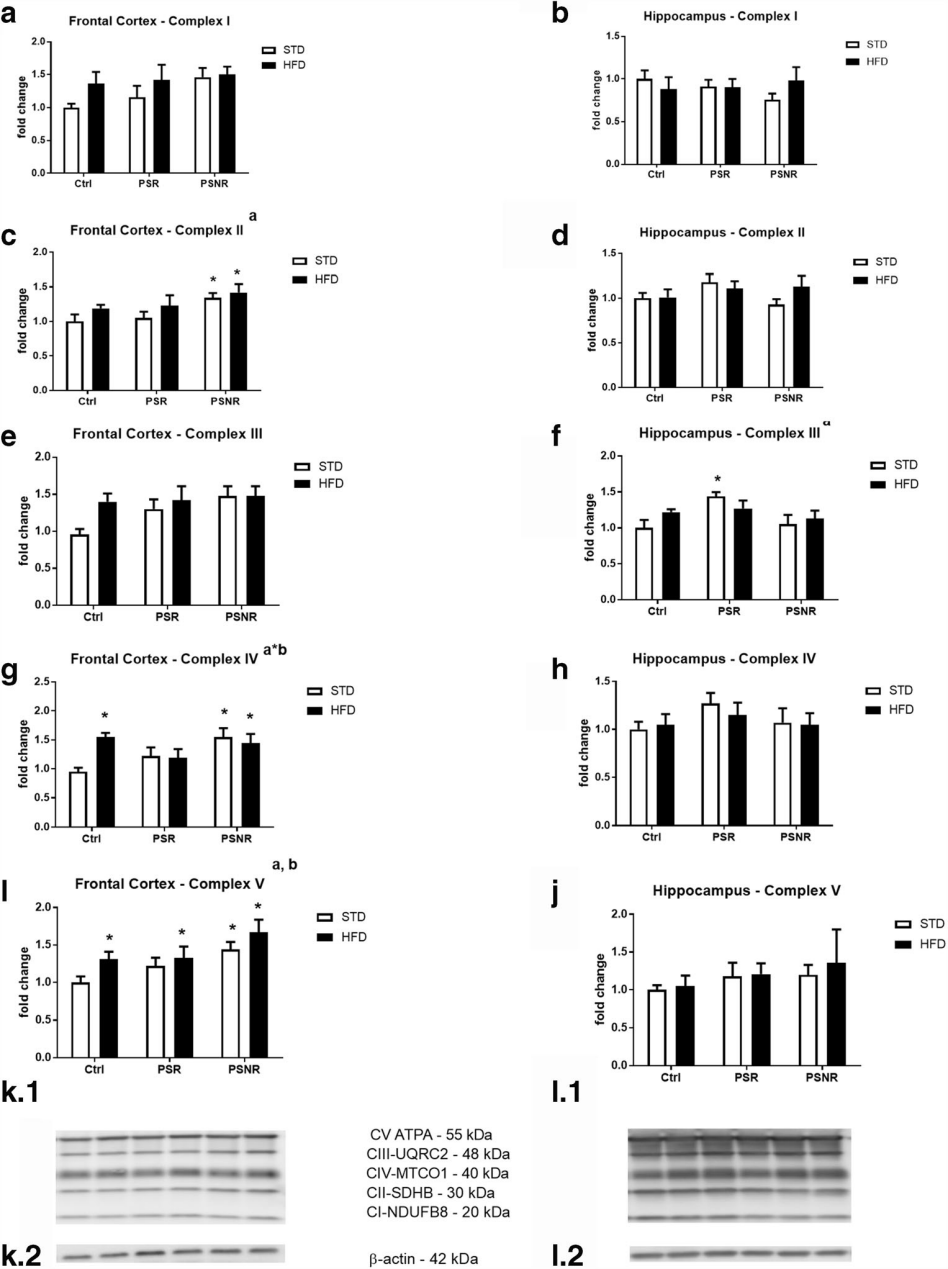
The effect of prenatal stress and high-fat diet on the expression of complexes I – V in the frontal cortex (A, C, E, G, I) and hippocampus (B, D, F, H, J). (K.1/L.1) Representative immunoblots of OXPHOS complexes (frontal cortex, hippocampus, respectively). The bands from the left: (1) Ctrl/STD; (2) Ctrl/HFD; (3) PS-R/STD; (4) PS-R/HFD; (5) PS-NR/STD; (6) PS-NR/HFD. (K2/L2) β-Actin as a loading control. The results are expressed as the means ± SEM. (a) statistically significant effect of prenatal stress; (b) statistically significant effect of HFD; (a*b) statistically significant effect of prenatal stress/HFD interaction. *p < 0.05 vs. control group (without prenatal stress, fed with STD). *n* = 6–8

There was no effect of prenatal stress, HFD, or the PS/HFD interaction on the expression of complexes I and III in the frontal cortex but a significant effect of PS (*F*_2,36_ = 3.71, *p* = 0.0343) on complex II, an effect of PS (*F*_2,35_ = 5.60, *p* = 0.0078) and HFD (*F*_1,35_ = 5.24, *p* = 0.0282) on complex V and an effect of the PS/HFD interaction (*F*_2,34_ = 3.84, *p* = 0.0312) on complex IV (Fig. 8A, C, E, G, I) were observed. Post hoc analysis showed an increase in complex II expression in PS-NR rats fed with a STD (*p* = 0.0417) and HFD (*p* = 0.0265), increase in complex IV in the control/HFD group (*p* = 0.0150) and in PS-NR rats fed with a STD (*p* = 0.0061) and HFD (*p* = 0.0277), and elevated expression of complex V in the control/HFD group (*p* = 0.0439), PS-R/HFD (*p* = 0.0328), and PS-NR groups fed with a STD (*p* = 0.0114) and HFD (*p* = 0.0010). In the second examined brain structure, the hippocampus, ANOVA revealed that PS exerted an effect (*F*_2,39_ = 4.11, *p* = 0.0241) only on complex III expression, while no effect of the HFD or PS/HFD interaction on the level of any complex was observed (Fig. 8B, D, F, H, J). Post hoc test showed an increase in complex III expression in PS-R rats fed with a STD (*p* = 0.0111).

### PS Increased While HFD Lowered the OXPHOS State Activity

Because PS and HFD influenced expression of respiratory chain complexes mainly in the frontal cortex, mitochondrial respiratory function was determined in this brain region.

To characterize mitochondrial function, the oxygen consumption rate (OCR) was determined at selected steps of oxidative phosphorylation in isolated mitochondrial fractions of the frontal cortex.

**Fig. 9:**
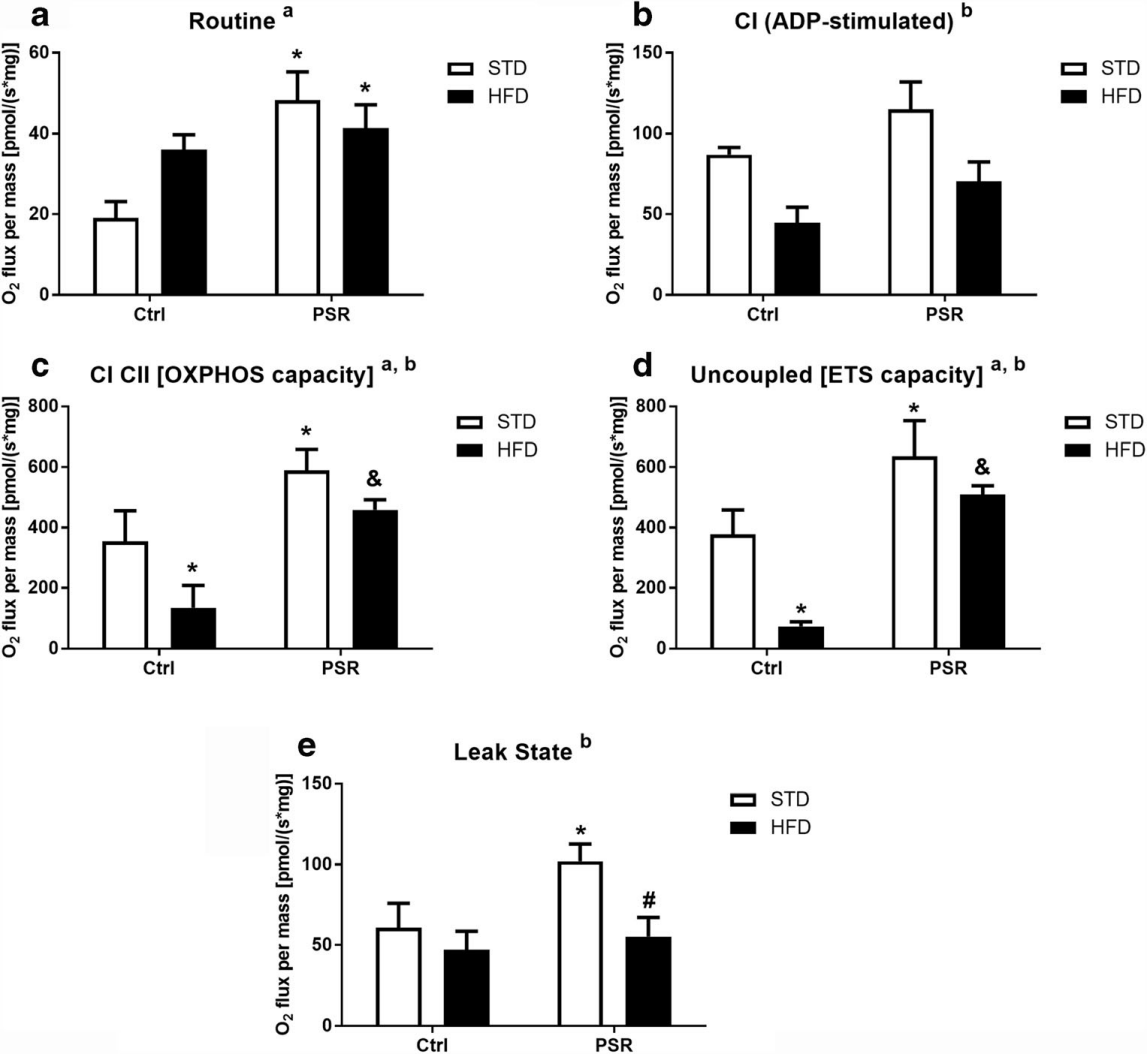
The effect of prenatal stress and high-fat diet on the oxygen consumption rate (OCR) in mitochondria isolated from the frontal cortex. Routine state (**A**). ADP-stimulated respiration (**B**). OXPHOS capacity (**C**). Uncoupled respiration measured in the presence of FCCP (**D**). Leak respiration measured in the absence of ADP (**E**). The results are expressed as the means ± SEM. (a) Statistically significant effect of prenatal stress; (b) statistically significant effect of HFD. *p < 0.05 vs. control group (without prenatal stress, fed with STD); ^#^*p* < 0.05 vs. appropriate STD group; ^&^*p* < 0.05 vs. control group (without prenatal stress, fed with HFD). *n* = 2–6

Using ANOVA, it has been found that PS affected the routine state (*F*_1,11_ = 6.92, *p* = 0.0234), and the Duncan test showed a significant increase in both PS-R/STD (*p* = 0.0135 ) and PS-R/HFD (*p* = 0.0431 ) groups (Fig. 9A). In the ADP-stimulated state, the effect of HFD was observed (*F*_1,11_ = 8.67, *p* = 0.0186); however, post hoc test did not show significant differences between any of the groups (Fig. 9B).

Both investigated factors, PS (*F*_1,13_ = 15.92, *p* = 0.0015) and HFD (*F*_1,13_ = 6.23, *p* = 0.0267), affected the OXPHOS state (after succinate addition) and uncoupled state (evaluated in the presence of FCCP) (*F*_1,13_ = 24.86 PS, *p* = 0.0002 and *F*_1,13_ = 9.57 HFD, *p* = 0.0086) (Fig. 9C, D). A significant increase in the OXPHOS capacity and uncoupled state in the PS-R/STD groups in comparison to the control/STD rats (for OXPHOS, *p* = 0.0409; for uncoupled state, *p* = 0.0259), decrease in the control/HFD in comparison to the control/STD (for OXPHOS, *p* = 0.0458; for uncoupled, *p* = 0.0090) and difference between the control/HFD and PS-R/HFD groups (for OXPHOS, *p* = 0.0080; for uncoupled, *p* = 0.0010) were shown by post hoc analysis.

In the leak state measured in the absence of ADP, ANOVA revealed only an effect of HFD (*F*_1,14_ = 5.72, *p* = 0.0314), but post hoc test showed an increase in the PS-R/STD animals in relation to control/STD group (*p* = 0.0369) and significant decrease in PS-R/HFD group vs. PS-R/STD (*p* = 0.0257) (Fig. 9E).

### PS and HFD Had No Significant Effect on ATP Synthesis in the Frontal Cortex and Hippocampus

ATP concentration was assayed in order to determine whether changes in the expression and function of mitochondrial complexes influenced its synthesis.

No significant effect of prenatal stress, the HFD, and their interaction on the ATP concentration in the frontal cortex or hippocampus was observed (Table 1).

**Table 1:** The effects of prenatal stress and 16 weeks on a high-fat diet on the level of ATP in the frontal cortex and hippocampus

ATP (nmol/g protein)	Control		PS reactive		PS nonreactive	
	STD	HFD	STD	HFD	STD	HFD
Frontal cortex	375.6 ± 34.94	367.4 ± 47.43	385.0 ± 36.45	405.4 ± 52.77	444.0 ± 7.92	380.8 ± 26.08
Hippocampus	408.0 ± 50.60	314.2 ± 24.96	347.3 ± 34.28	367.4 ± 37.26	306.9 ± 50.53	261.8 ± 37.85

The results are presented as nmol/g protein and expressed as the means ± the SEM from 8 animals per group

### No Effect of PS and HFD on UCP4 Level in the Frontal Cortex and Hippocampus

Increased oxygen consumption rate in the leak state in prenatally stressed rats suggested uncoupling of oxidative phosphorylation from ATP synthesis; therefore, expression of uncoupling protein (UCP4) was determined.

No significant effect of prenatal stress, the HFD, or their interaction on the UCP4 level in the frontal cortex or hippocampus was detected (Table 2).

**Table 2:** The effects of prenatal stress and 16 weeks on a high-fat diet on the level of UCP4 in the frontal cortex and hippocampus

UCP4 (fold change)	Control		PS reactive		PS nonreactive	
	STD	HFD	STD	HFD	STD	HFD
Frontal cortex	1.00 ± 0.09	0.59 ± 0.06	0.85 ± 0.08	1.11 ± 0.20	0.94 ± 0.14	0.90 ± 0.13
Hippocampus	1.00 ± 0.10	0.92 ± 0.16	1.01 ± 0.18	0.97 ± 0.20	1.19 ± 0.15	0.82 ± 0.15

The results are presented as fold of change and expressed as the means ± the SEM from 7–8 animals per group

### PS Increased the Level of Insulin in the Frontal Cortex, but Decreased Its Concentration in the Hippocampus

Insulin is major hormone regulating glucose metabolism in the periphery and in the brain in addition to the neuroprotective effect also can affect metabolism; therefore, insulin, insulin receptor expression, and Akt phosphorylation in the frontal cortex and hippocampus were studied.

**Fig. 10:**
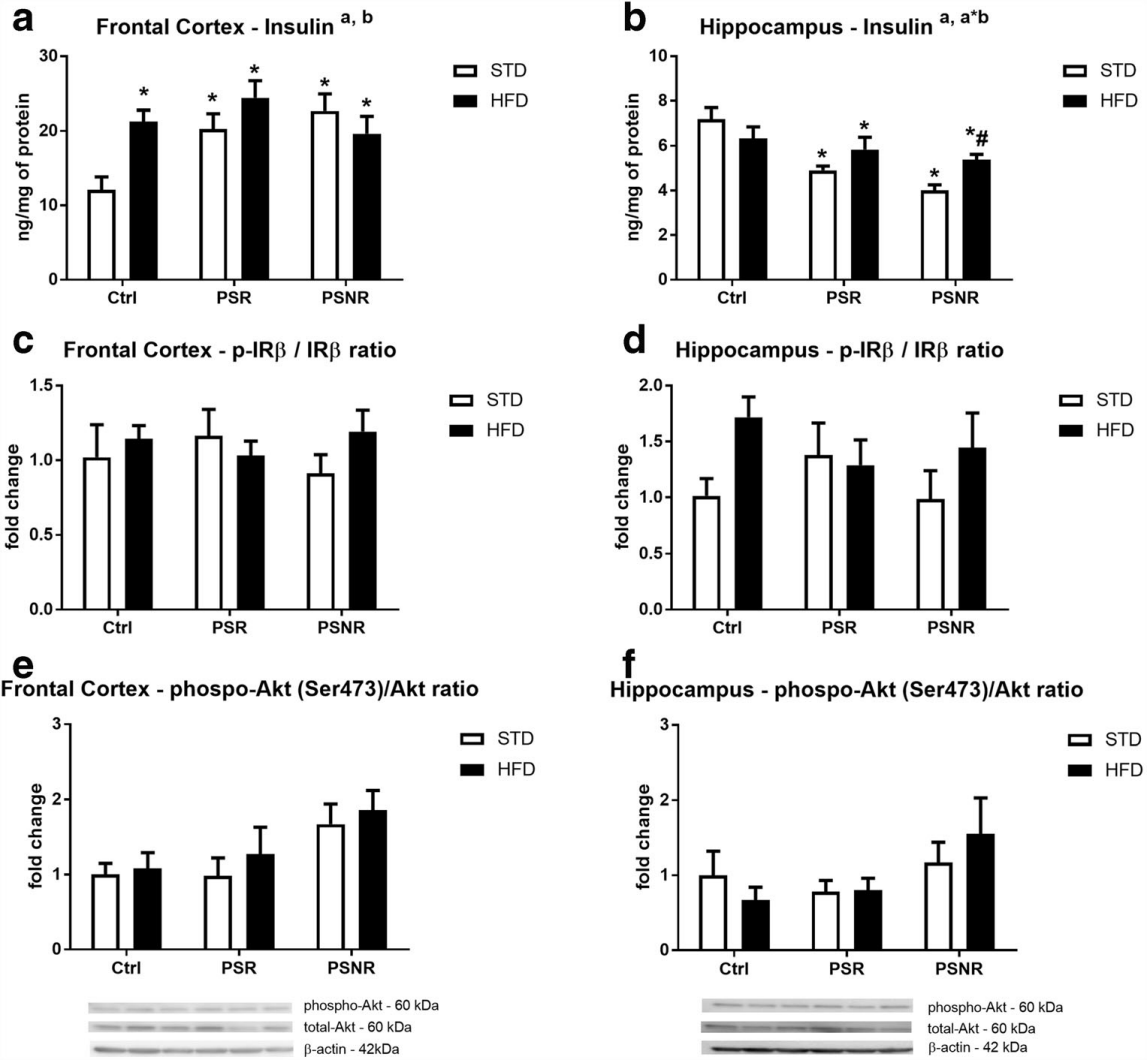
The effect of prenatal stress and high-fat diet on the insulin concentration in the frontal cortex (**A**) and hippocampus (**B**), on the ratio of active to inactive forms of the insulin receptor (IR) in the frontal cortex (**C**) and hippocampus (**D**), and on the ratio of phospho-Akt (Ser473)/Akt in the frontal cortex (**E**) and hippocampus (**F**). The bands from the left: (1) Ctrl/STD; (2) Ctrl/HFD; (3) PS-R/STD; (4) PS-R/HFD; (5) PS-NR/STD; (6) PS-NR/HFD. β-Actin as a loading control (**E**, **F**). The results are expressed as the means ± SEM. (a) Statistically significant effect of prenatal stress, (b) statistically significant effect of HFD, (a*b) statistically significant effect of prenatal stress/HFD interaction. *p < 0.05 vs. control group (without prenatal stress, fed with STD); #*p* < 0.05 vs. appropriate STD group. *n* = 6–10 (**A**–**D**), *n* = 4–6 (**E**–**F**)

Prenatal stress influenced insulin levels in the frontal cortex and hippocampus in an inverse way. We showed a significant effect of PS (*F*_2,51_ = 3.78, *p* = 0.0296) and HFD (*F*_1,51_ = 4.40, *p* = 0.0408) on insulin concentration in the frontal cortex, and further analysis indicated an increase in this hormone level in all experimental groups in comparison to the control/STD rats (control/HFD: *p* = 0.0095; PS-R/STD: *p* = 0.0131; PS-R/HFD: *p* = 0.0008; PS-NR/STD: *p* = 0.0034; PS-NR/HFD: *p* = 0.0104 ) (Fig. 10A). In the hippocampus, PS (*F*_2,42_ = 13.86, *p* < 0.0001) and the PS/HFD interaction (*F*_2,42_ = 4.38, *p* = 0.0188) affected insulin levels (Fig. 10B). Moreover, post hoc analysis showed that in all animals subjected to prenatal stress, both those receiving the STD and HFD, levels of this hormone were lower than those in control/STD animals (PS-R/STD: *p* = 0.0005; PS-R/HFD: *p* = 0.0271; PS-NR/STD: *p* < 0.0001; PS-NR/HFD: *p* = 0.0047). Moreover, insulin level was significantly lower in PS-NR/STD group than in PS-NR/HFD (*p* = 0.0246). The ratio of phosphorylated to total receptor forms (p-IR/IR) in the frontal cortex and hippocampus (Fig. 10C, D) did not differ between the groups. Furthermore, no significant effect of PS, the HFD, or their interaction on the ratio of phosphorylated to total Akt form in the frontal cortex or hippocampus was detected (Fig. 10E, F).

### PS and HFD Decreased GLP-1 Concentration in the Frontal Cortex but Not in the Hippocampus

In addition to insulin, GLP-1 is the second major hormone that regulates peripheral and possibly also brain metabolism, so its concentration and expression of its receptors in the frontal cortex and hippocampus were determined.

**Fig. 11:**
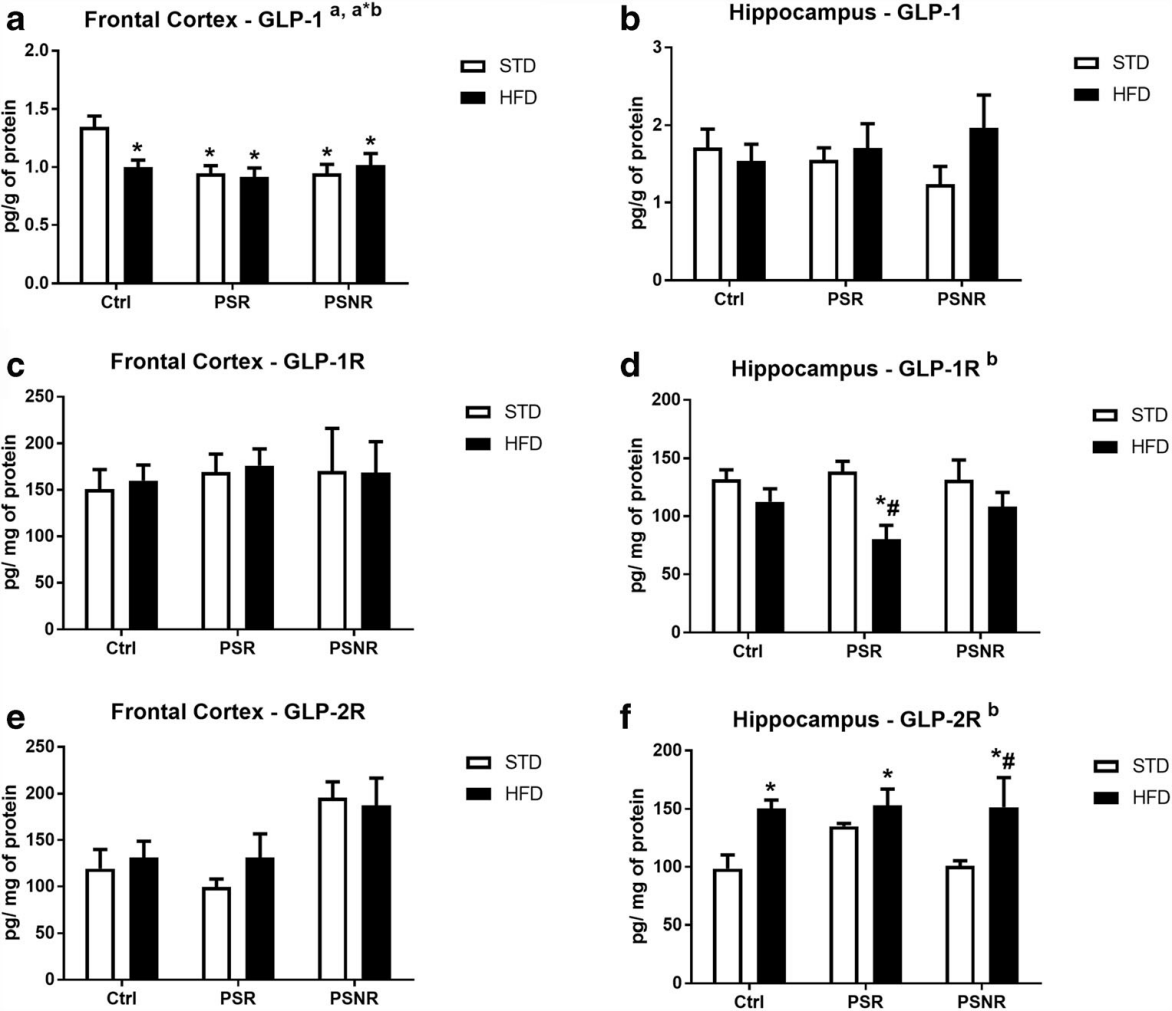
The effect of prenatal stress and high-fat diet on the expression of GLP-1 (**A**), GLP-1 receptor (**C**), and GLP-2 receptor (**E**) in the frontal cortex and on the expression of GLP-1 (**B**), GLP-1 receptor (**D**), and GLP-2 receptor (**F**) in the hippocampus. The results are expressed as the means ± SEM. (a) Statistically significant effect of prenatal stress; (b) statistically significant effect of HFD; (a*b) statistically significant effect of prenatal stress/HFD interaction. *p < 0.05 vs. control group (without prenatal stress, fed with STD); ^#^*p* < 0.05 vs. appropriate STD group. *n* = 7–14

ANOVA showed an effects of PS (*F*_2,61_ = 4.59, *p* = 0.0139) and PS/HFD interaction (*F*_2,61_ = 3.44, *p* = 0.0385) in the frontal cortex. Further analysis revealed a decrease in GLP-1 levels in all experimental groups in comparison to that in the control/STD animals (control/HFD: *p* = 0.0052; PS-R/STD: *p* = 0.0020; PS-R/HFD: *p* = 0.0012; PS-NR/STD: *p* = 0.0023; PS-NR/HFD: *p* = 0.0065 (Fig. 11A). However, none of the factors studied affected the level of GLP-1R and GLP-2R in the frontal cortex (Fig. 11C, E).

In the hippocampus, none of the factors studied (PS, HFD, and PS/HFD interaction) had an effect on the level of GLP-1. ANOVA showed an effect of HFD on GLP-1 receptor (GLP-1R) level (*F*_1,38_ = 10.43, *p* = 0.0026) and GLP-2 receptor GLP-2R (*F*_1,39_ = 12.09, *p* = 0.0013) (Fig. 11D, F) in the hippocampus. We observed a decrease in GLP-1R level in PS-R/HFD rats in comparison to control/STD (*p* = 0.0241) and PS-R/STD groups (*p* = 0.0133). In contrast to the GLP-1R receptor, the HFD increased the level of GLP-2R in control animals, PS-R and PS-NR animals fed with the HFD (control/HFD: *p* = 0.0317; PS-R/HFD: *p* = 0.0140; PS-NR/HFD: *p* = 0.0152). Moreover, we observed the increase in GLP-2R in PS-NR/HFD animals in comparison to PS-NR/STD (*p* = 0.0176) (Fig. 11F).

Table 3 displays the most important effects observed in selected markers. It contains the results from selected groups essential for the study described in the article.

**Table 3:** The summary of the most important effects observed in the chosen markers. The changes in selected groups of animals (in comparison to Ctrl/STD group) are shown

	Frontal cortex				Hippocampus			
	Ctrl/STD	Ctrl/HFD	PS-R/STD	PS-R/HFD	Ctrl/STD	Ctrl/HFD	PS-R/STD	PS-R/HFD
Glucose-6-phosphate		–	–	**↑**		**↑**	**↑**	**↑**
Glycogen		–	–	**↑**		–	**↑**	–
GLUT4-membrane		–	–	**↓**		–	–	–
PDH activity		–	–	–		**↓**	**↓**	**↓**
G-6-PD activity		–	**↑**	–		–	–	–
Insulin		**↑**	**↑**	**↑**		–	**↓**	**↓**
GLP-1		**↓**	**↓**	**↓**		–	–	–
GLP-1R		–	–	–		–	–	**↓**
GLP-2R		–	–	–		**↑**	–	**↑**
Complex III		–	–	–		–	**↑**	–
Complex IV		**↑**	–	–		–	–	–
Complex V		**↑**	–	**↑**		–	–	–
Oxygen consumption rate								
Routine respiration		–	**↑**	**↑**	Not tested			
OXPHOS capacity		**↓**	**↑**	–				
Uncoupled		**↓**	**↑**	–				
Leak state		–	**↑**	–				

## Discussion

The present study has shown that both prenatal stress and the HFD increased glucose-6-phosphate levels in the hippocampus, whereas in the frontal cortex, this effect was observed in prenatally stress-nonreactive animals, while in stress-reactive only in these receiving HFD. Although the metabolic processes in the brain are relatively poorly understood, glucose is recognized as the main source of energy in the brain. Glucose-6-phosphate is formed from glucose transported to cells and is a substrate in various metabolic pathways of carbohydrate metabolism. Increased concentrations of G-6-P in the hippocampus of prenatally stressed animals and in the frontal cortex in PS-R rats fed with HFD suggested that in the depression or depression with obesity models used, glucose uptake into brain cells was enhanced or its metabolism was slower. These data are in line with our previous results, which showed an increased hippocampal glucose level in prenatally stressed 3-month-old rats (Detka et al. 2014). Some quantities of G-6-P may be formed in the glycogenolysis process from glycogen stored in astrocytes; however, since prenatal stress enhanced glycogen content in the hippocampus of PS-R rats and in the frontal cortex in PS-R animals fed with HFD, such a possibility could be excluded. Although functional consequences of increased glycogen storage in the brain structures are difficult to predict, its increased accumulation is often associated with reduced neuronal activity. Inhibition of glycogen metabolism has been shown to reduce the formation of miniature postsynaptic stimulating potentials in co-cultures of neurons and astrocytes (Mozrzymas et al. 2011) and also contributed to the impairment of processes related to memory consolidation in the hippocampus of rodents (Newman et al. 2011). The high-fat diet also increased G-6-P levels in the hippocampus and enhanced the prenatal stress effect on this glucose metabolite only in prenatal stress-nonresponsive rats and not in those animals in which stress-induced depression-like behavior. Increased levels of G-6-P and glycogen in the frontal cortex of PS-NR rats may to some extent result from the increased expression of the GLUT1 transporter, which in the hippocampus and frontal cortex occurs in much higher concentration than GLUT3 and GLUT4 (Detka et al. 2014), and it has been shown that some factors producing adverse effects on the brain increase its concentration (Gerhart et al. 1994; Carver et al. 1999). However, this relationship was only seen in PS-NR animals fed a high-fat diet in which an increased GLUT1 level in the frontal cortex was accompanied by elevated glycogen and G-6-P expression. It is difficult to explain why the increase in the concentrations of G-6-P and glycogen or expression of mitochondrial complex V was more visible in the frontal cortex in stress-nonreactive than stress-reactive animals. It is known, that not all animals respond to some stress procedures used in animal models of depression with behavioral depression-like symptoms, but they predominantly cause similar biochemical changes (Kolasa et al. 2014; Kurek et al. 2018). Maybe some of these changes are adaptive and delay the onset of pro-depressive behavior.

Assessing the process of glycolysis, we showed that prenatal stress and HFD did not affect either of the key enzymes involved in this process—phosphofructokinase or the main brain metabolite, lactate in responsive animals. This is consistent with our previous studies in which we did not observe an effect of prenatal stress on these markers under the basal conditions; however, acute stress and/or glucose load increased the expression of these markers in the frontal cortex of prenatally stressed animals (Detka et al. 2015), which proves that prenatal stress intensifies the glycolysis process, but only in response to additional stressors applied in the adulthood.

The next process of glucose metabolism, i.e., transformation of pyruvate into acetyl-CoA by the pyruvate dehydrogenase complex, seems to be weakened in the hippocampus by prenatal stress and HFD. Lower activity of this enzyme was observed in control animals fed a high-fat diet and PS-R rats fed with both standard and high-fat diet; however, the HFD did not exacerbate changes in the activity of this enzyme induced by prenatal stress. Interestingly, pyruvate dehydrogenase activity in the hippocampus of PS-NR rats did not significantly differ from the activity of this enzyme in control animals. In the depression model used, similar to other animal models of depression, despite the action of identical stress factors, behavioral depression-like changes were present in some (PS-R) but not all animals, and so far, the cause of these differences has not been explained. Maybe one of the reasons is a weaker inhibitory effect of stress factors on the activity of pyruvate dehydrogenase in the hippocampus. The reduced activity of pyruvate dehydrogenase observed only in the hippocampus and not in the frontal cortex could have resulted from the differences in insulin concentration in these two brain structures; prenatal stress decreased the level of insulin in the hippocampus, while in the frontal cortex, both stress and HFD increased the concentration of this hormone. Insulin increases the activity of pyruvate dehydrogenase in peripheral tissues, and although this action in the brain was not investigated, it cannot be excluded.

Intensification by prenatal stress of almost all measured steps of oxidative phosphorylation and enhanced expression of some mitochondrial complexes in the frontal cortex confirmed the increased oxidative phosphorylation in this brain structure. However, prenatal stress had no effect on ATP synthesis, which suggested reduced mitochondrial respiration efficiency. Because prenatal stress enhanced the oxygen consumption rate in the leak state when oxygen flux is maintained mainly to compensate for the proton leak when ATP synthase is not active, this suggested that in prenatally stressed rats fed with STD, the inner mitochondrial membrane was more permeable to protons and as a consequence, uncoupling of oxidative phosphorylation from ATP synthesis may occur and thus reduce ATP synthesis by complex V. Increased expression of some mitochondrial complexes and activity of the OXPHOS state induced by prenatal stress could be a compensatory mechanism activated in response to the decrease in mitochondrial ATP production. Then we checked whether the process of uncoupling oxidative phosphorylation from ATP synthesis may depend on overexpression of uncoupling protein 4 (UCP4). We did not observe an increase in UCP4 levels in the frontal cortex, which indicated that this protein was not involved in prenatal stress-induced metabolic changes. However, the process of oxidative phosphorylation uncoupling in the brain can also be regulated by other factors such as UCP5, insulin-like growth factor, and mitochondrial fusion proteins (Liu et al. 2006; Logan et al. 2018).

Because the level of ATP in the frontal cortex was not reduced by prenatal stress, it seemed, therefore, that the increase in oxidative phosphorylation was sufficient to maintain the production of ATP in basal condition. However, under stress conditions, it is possible that there is a shift of the amount of ATP synthesized in the frontal cortex from oxidative phosphorylation to glycolytic processes, because previously we found that prenatal stress increased the expression of the key enzyme of glycolysis—phosphofructokinase—and the main product of glycolysis—lactate—in the frontal cortex of rats subjected to stress in adulthood (Detka et al. 2015). Such a shift in the mode of ATP production was previously observed in pheochromocytoma (PC12) cell lines (Liu et al. 2006). The HFD increased the expression of complexes IV and V in the frontal cortex in control rats; however, it inhibited OXPHOS and uncoupled states in control animals. It seems, therefore, that HFD affected the permeability of the mitochondrial membrane in a way opposite to that of prenatal stress; however, this effect did not translate into an increase in ATP synthesis.

In this study, we did not evaluate the activity of particular steps of oxidative phosphorylation in the hippocampus, so it is impossible to tell whether similar changes may also occur in this brain structure. However, our study showed that prenatal stress also raised the expression of complex III in this structure.

Many data have shown that the HFD differentially affects the effects of stress (Finger et al. 2011). In the present study, the HFD intensified the effects of prenatal stress only on glucose-6-phosphate concentration in the hippocampus in PS-NR rats, on GLUT1 expression in the frontal cortex in PS-NR rats and on the decrease in the membrane GLUT4 in the frontal cortex in PS-R animals. In contrast, the HFD weakened some of the prenatal stress-induced effects, such as the decrease in lactate and insulin levels in the hippocampus of PS-NR rats and the increase in the oxygen consumption rate in the OXPHOS, uncoupled and leak states of oxidative phosphorylation in the frontal cortex. It should be noted that although in the present investigation, effectiveness of the HFD was checked only by demonstrating its effect on the weight gain, whereas in our previous study, conducted in animals treated in the same way, we found that HFD significantly increased blood insulin levels (Kurek et al. 2018).

Because insulin and GLP-1 are fundamentally involved in the regulation of glucose metabolism in the periphery and it is known that these hormones also affect brain metabolism (Detka et al. 2013; Bassil et al. 2014), their concentrations were measured. In the frontal cortex, both prenatal stress and the HFD increased insulin levels; however, a decrease in the membrane glucose transporter (GLUT4) observed in the PS-R/HFD rats suggested weaker insulin action in the model of coexistence of depression and obesity. The same data showed that glucocorticoids caused insulin resistance not only in the periphery but also in the brain, but so far it has been demonstrated mainly in the hypothalamus (Pan et al. 2013). Diminished level of membrane GLUT4 in the frontal cortex of PS-R/HFD group and higher insulin concentration than in the control animals suggested that in animals in which prenatal stress caused depression-like symptoms and which additionally received a high-fat diet in adulthood, insulin resistance in the frontal cortex may develop. However, this suggestion can only be confirmed by demonstrating such a change in animals after insulin administration. Moreover, decreased membrane GLUT4 level was not associated with the reduction of p-IR and p-Akt levels—PS and HFD did not change concentrations of p-IR/IR and p-Akt/Akt ratio. It is possible that weaker insulin effect in the frontal cortex in PS-R/HFD rats resulted from the disturbance in a further stage of intracellular insulin pathway.

Both prenatal stress and the HFD decreased the frontocortical levels of GLP-1, a hormone which in addition to the effect on metabolism in the periphery and brain exerts a number of neuroprotective effects, and this may be the reason why our previous studies indicate that prenatal stress causes more adverse changes in the frontal cortex than in the hippocampus (Kucharczyk et al. 2018) in contrast to the majority of data indicating that the hippocampus is the brain structure most sensitive to damage. In the hippocampus, the level of GLP-1 did not change under the influence of prenatal stress and the HFD, but in this structure, the HFD caused a decrease in expression of its receptors, however, at the same time, there was a compensatory increase in the expression of the receptor for GLP-2, which, like GLP-1, exerts an anorectic and neuroprotective effect.

Some of the changes observed in the present study in animals fed a HFD are similar to those reported in obese people. For example, our research has shown that HFD decreased some state of oxidative phosphorylation in the frontal cortex and similar changes were found in skeletal muscle of type 2 diabetic patients (Mogensen et al. 2007). Moreover, in the current studies, HFD decreased the level of GLP-1 in the frontal cortex and in obese people, and plasma level of this hormone was also lower than in controls (Hussein et al. 2014). Similar effect on GLUT1 transporters to that which we observed in the frontal cortex of PS-NR rats fed a HFD was detected in fibroblast and skeletal muscles of obese people (Miele et al. 1997).

Some PS- or HFD-induced changes were dependent on the brain structure studied. A decrease in pyruvate dehydrogenase activity, indicating a weakening of the Krebs cycle, was observed only in the hippocampus, while a decrease in the GLUT4 transporter in the membrane fraction was seen only in the frontal cortex. Among markers related to neurodegeneration/neuroprotection processes, the changes occurred mainly in the frontal cortex, but it is difficult to predict their consequences, because prenatal stress increased G6PD activity in PS-R/STD animals, suggesting protective effect, but on the other hand both PS and HFD strongly reduced the level of the neuroprotective peptide—GLP-1. However, it appears that the changes observed in the frontal cortex did not lead to disturbance in short-term memory processes, because previously we found no effect of prenatal stress and HFD on cortex-dependent short-term memory determined in the novel object recognition test (Kurek et al. 2018).

In conclusion, the obtained results indicated that not all effects observed after prenatal stress and high-fat diet could be considered as adverse changes; they depended on the brain structure studied and some PS- and HFD-induced effects were opposite. Most of the observed changes appear to be unfavorable; however, PS-induced increase in G6PD activity in the frontal cortex maybe propitious because this enzyme is essential for the generation of NADPH and protection against oxidative stress-induced cellular damage. Also HFD-induced increase in GLP-2 receptor level in hippocampus, which may increase neuroprotective GLP-1 action, seems to be beneficial. Among the changes that may be relevant in the case of coexistence of depression and obesity, the increase in the level of G-6-P, glycogen, and reduction of the membrane GLUT4 in the frontal cortex seem to be particularly important, because these changes were present only in PS-R/HFD group, but not in control/HFD or PS-R/STD. Among these changes, the decreased GLUT4 expression in the membrane fraction of the frontal cortex suggested weaker insulin action only in the case of co-occurrence of depression and obesity. On the other hand, since obesity and prenatal stress induced opposite changes in the oxygen consumption rate in the frontal cortex, these changes were less pronounced in comorbidity of depression and obesity. In the hippocampus, in the model of the co-occurrence of depression and obesity, the decreased in GLP-1 receptor suggests that neurodegenerative changes may be more severe in the case of coexistence of the two diseases compared to those observed in obesity or in depression.

## Abbreviations

BSA: Bovine serum albumin
FCx: Frontal cortex
G-6-P: Glucose-6-phosphate
G6PD: Glucose-6-phosphate dehydrogenase
GLP-1: Glucagon-like peptide-1
GLP-1R: Glucagon-like peptide-1 receptor
GLP-2: Glucagon-like peptide-2
GLP-2R: Glucagon-like peptide-2 receptor
GLUT: Glucose transporter
HFD: High-fat diet
Hp: Hippocampus
HPA: Hypothalamic–pituitary–adrenal axis
OCR: Oxygen consumption rate
OXPHOS: Oxidative phosphorylation
PDH: Pyruvate dehydrogenase
PFK-1 L: Phosphofructokinase
PS-NR: Prenatal stress-nonreactive
PS-R: Prenatal stress-reactive
SDS: Sodium dodecyl sulfate
UCP4: Uncoupling protein 4
